# Child Maltreatment and The Psychological Consequences of COVID-19 for Older Adults

**DOI:** 10.1093/geroni/igab046.3574

**Published:** 2021-12-17

**Authors:** Tyler Bruefach, Dawn Carr, Natalie Sachs-Ericsson

**Affiliations:** Florida State University, Tallahassee, Florida, United States

## Abstract

Traumatic experiences in early life impact adults’ well-being and their abilities to respond to adversities over the life course. Child maltreatment is a particularly salient stressor in childhood and scholars have noted the psychological implications of such experiences that extend into late life. People who experienced maltreatment in childhood have more difficulty maintaining and developing high quality relationships, regulating their emotions, and they engage in poorer coping behaviors amidst major stressors. Our study focuses on how child maltreatment (i.e., emotional abuse; physical abuse; sexual abuse; emotional neglect) shaped older adults’ changes in depression during the early stages of the COVID-19 Pandemic. Using a dataset released in 2021, based on a community sample of older adults collected in September 2018 and June 2020, we found that exposures to emotional neglect (1.630; p < 0.001) and emotional abuse (0.670; p < 0.05) in childhood were both associated with increases in depression scores in association with the pandemic, relative to those without such exposures. In addition, the more forms of maltreatment that individuals were exposed to in childhood, the more they experienced negative psychological health consequences in association with the pandemic. Our results suggest that early life traumas play a role in how older adults respond to stressful situations. Clinical treatments for depression may be more effective if they take into consideration how these early life experiences influence exposures to new stressors in later life.

